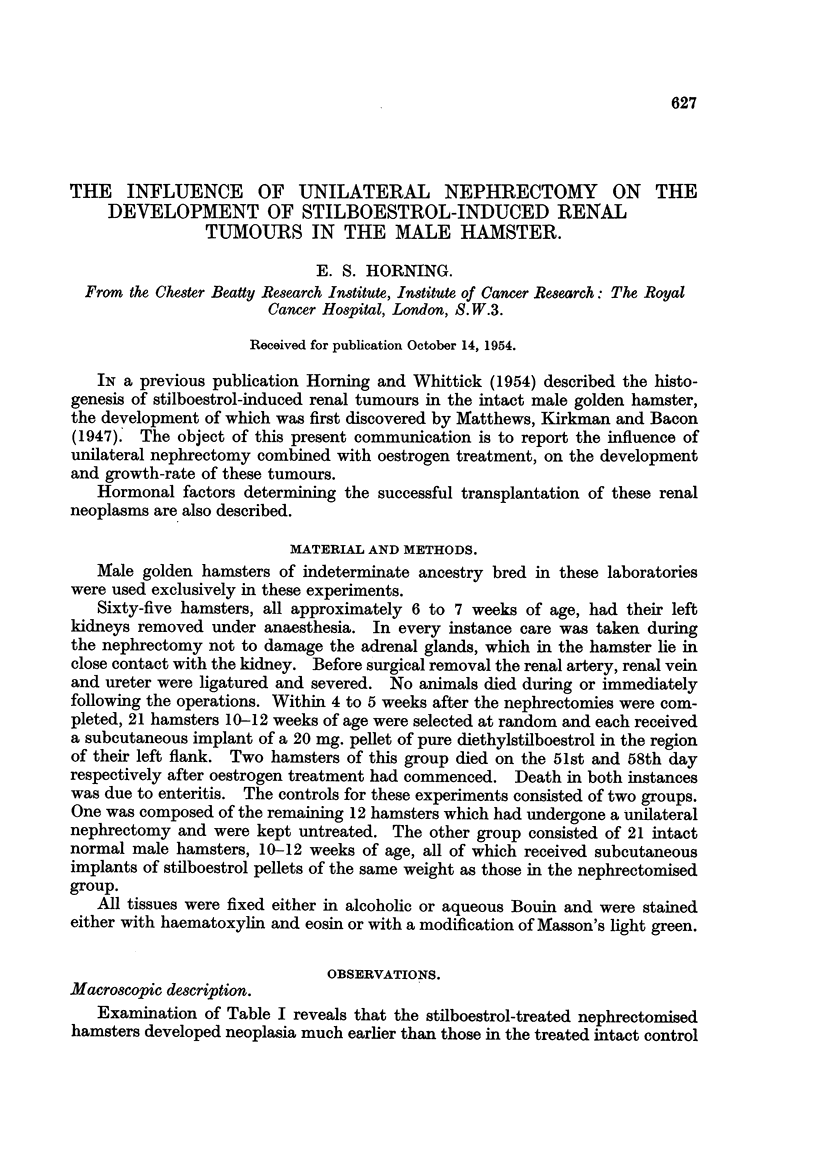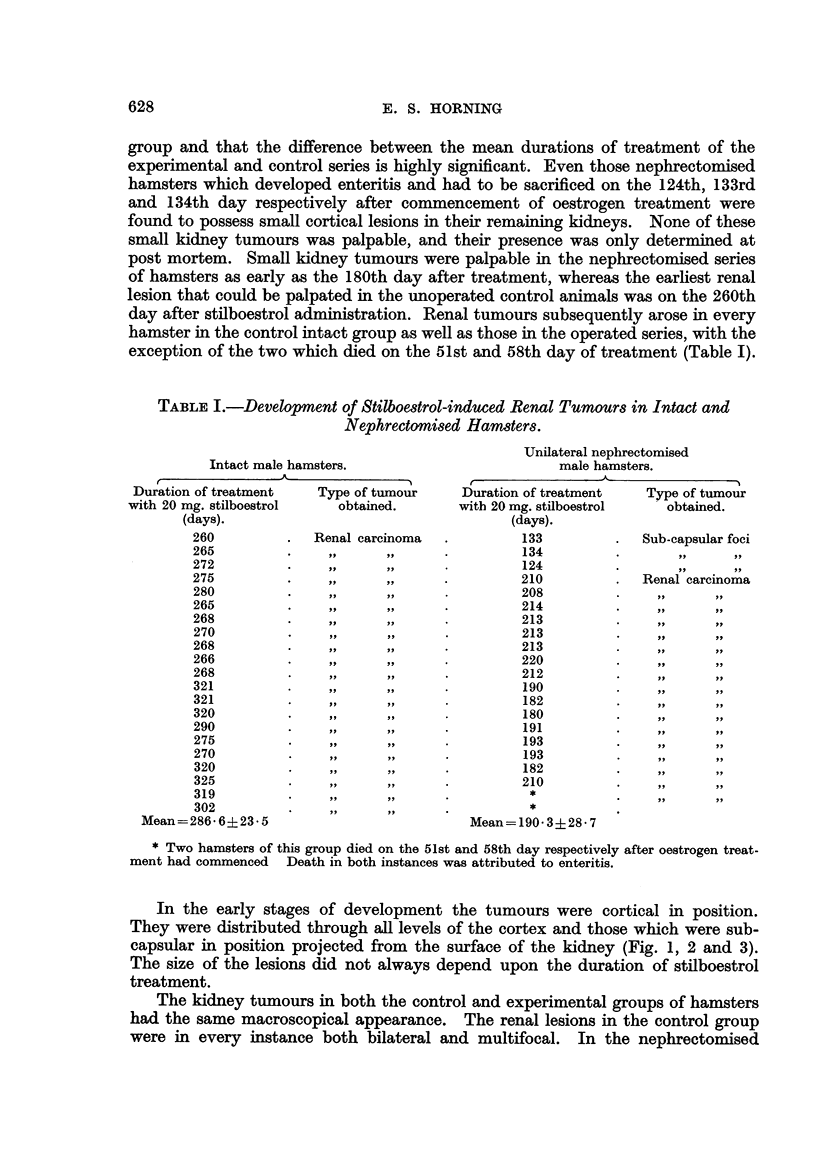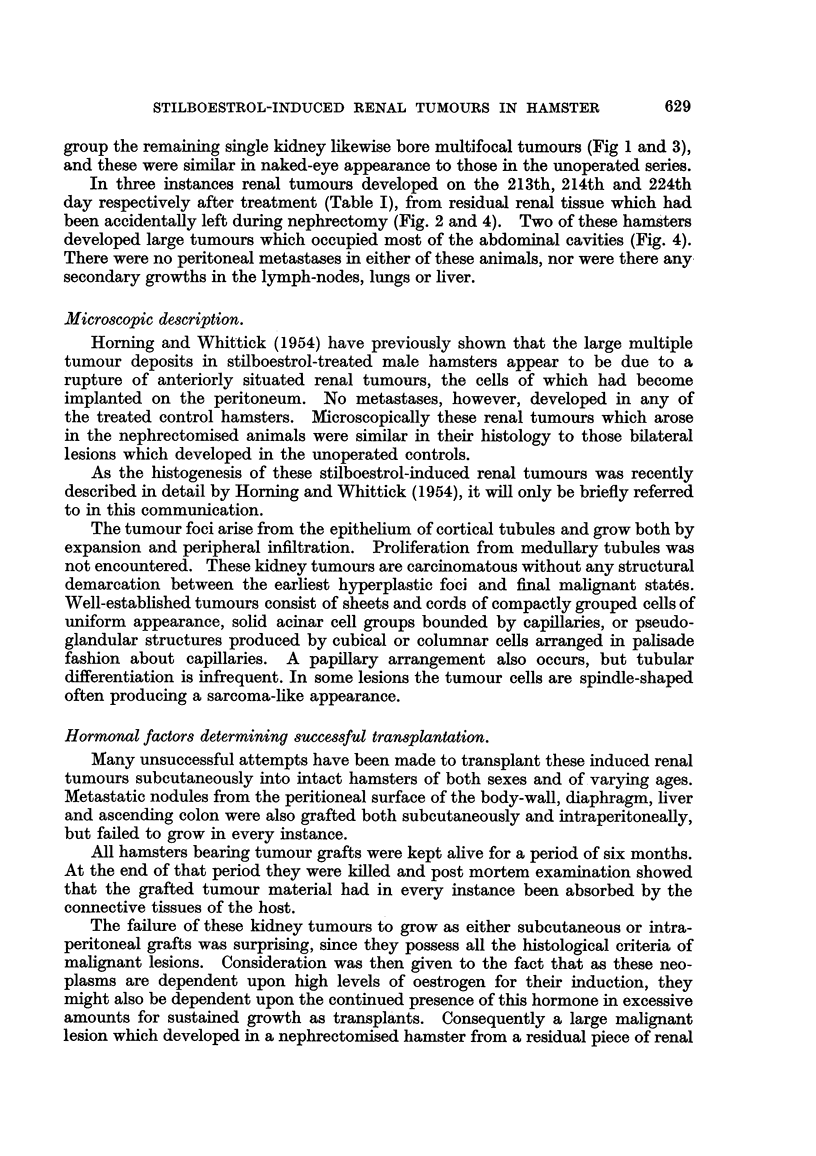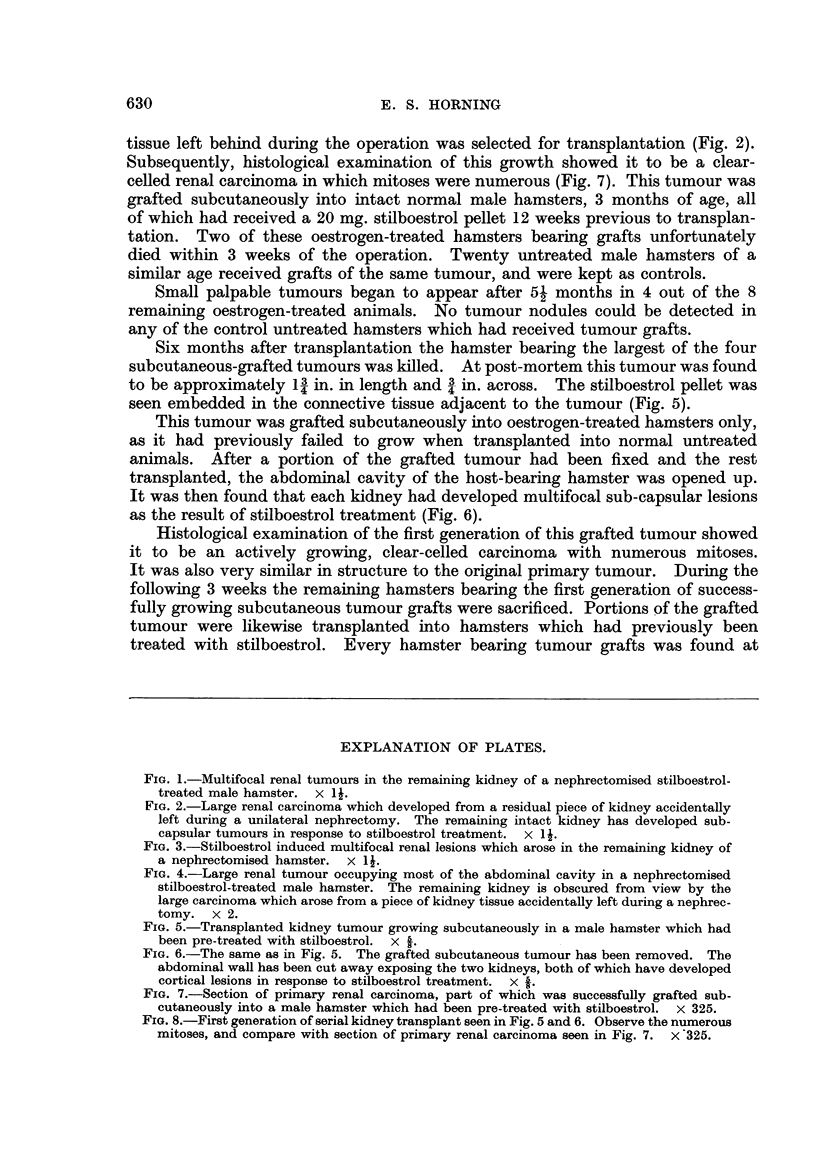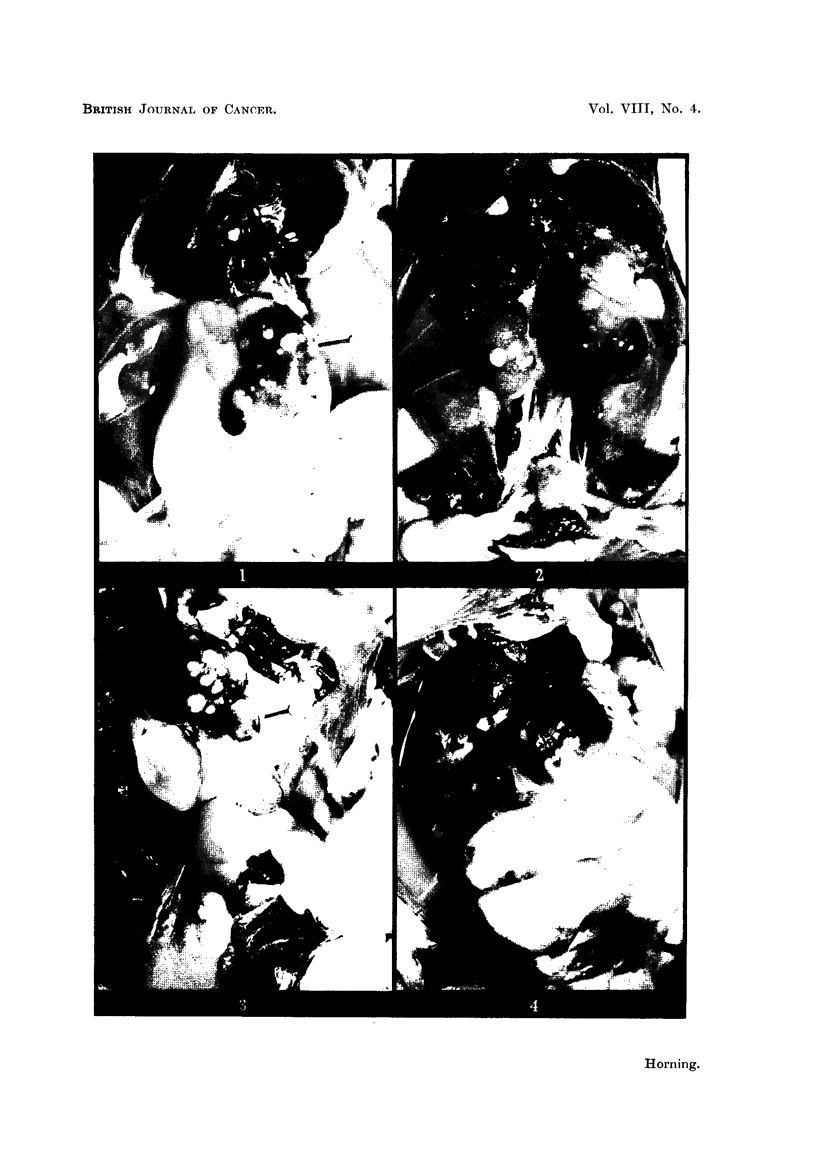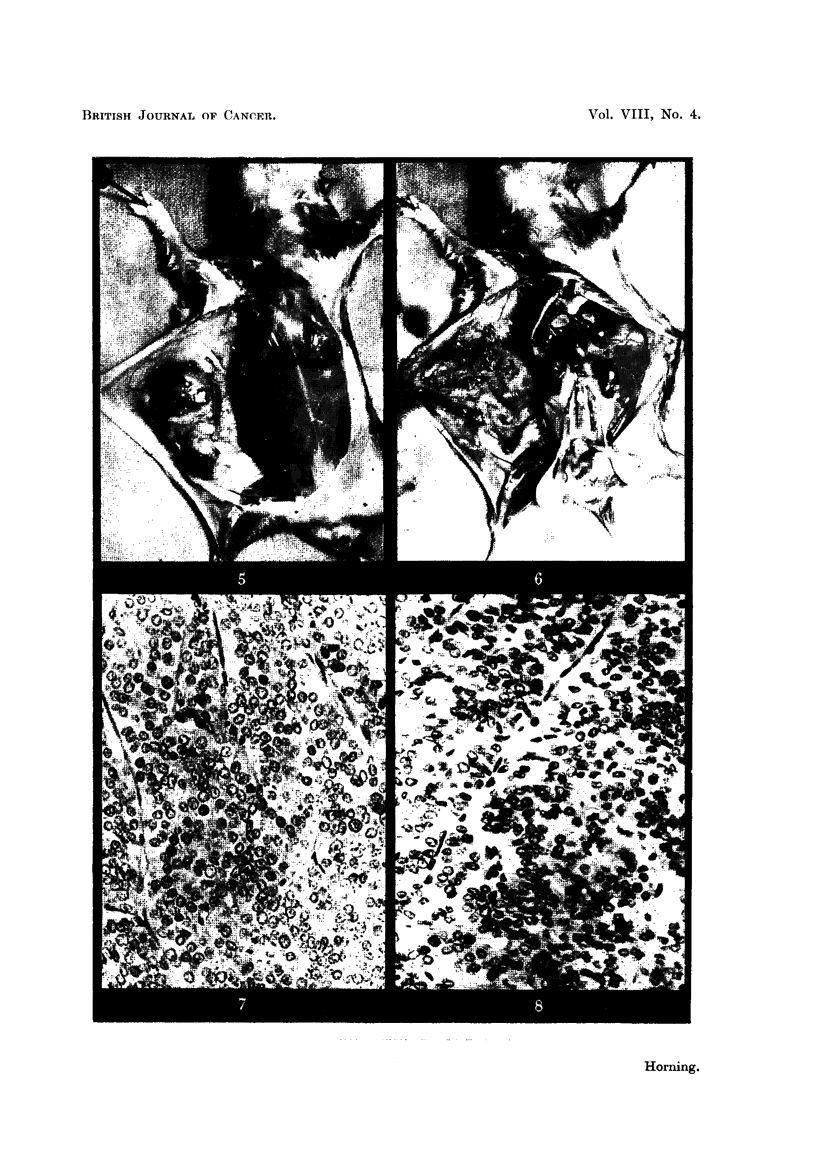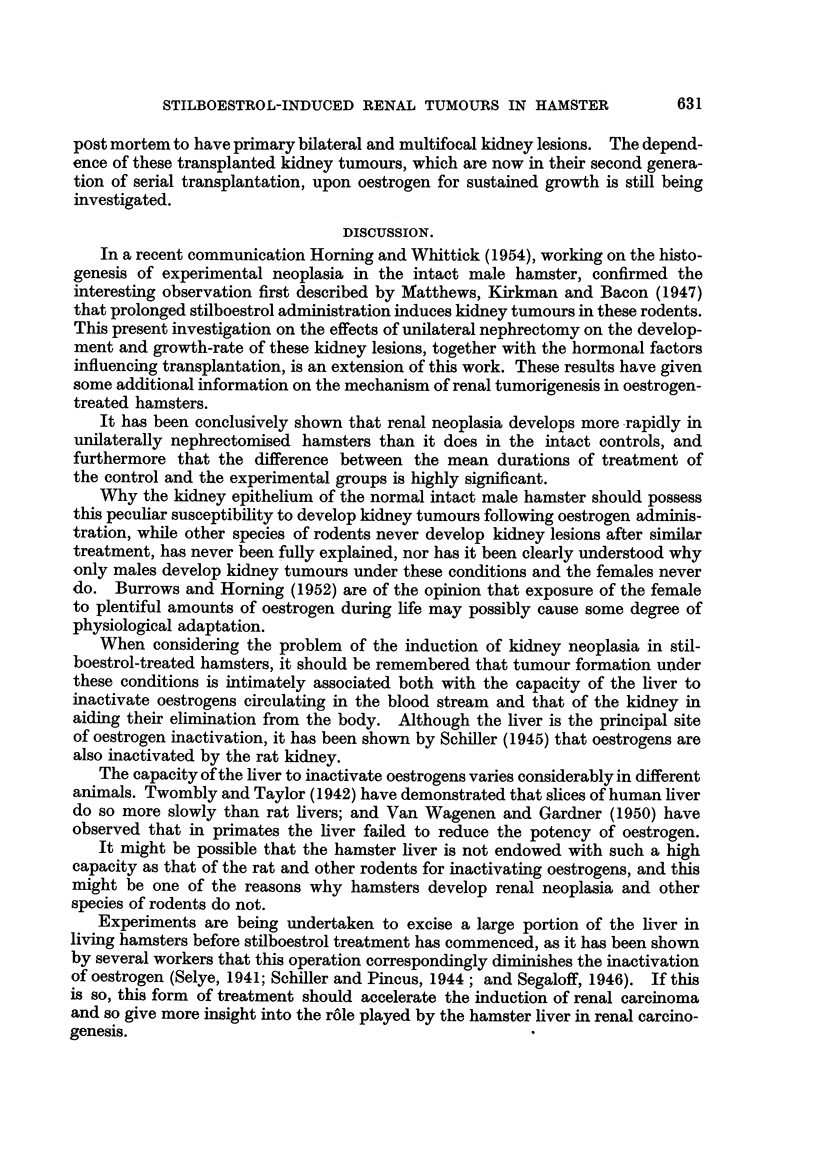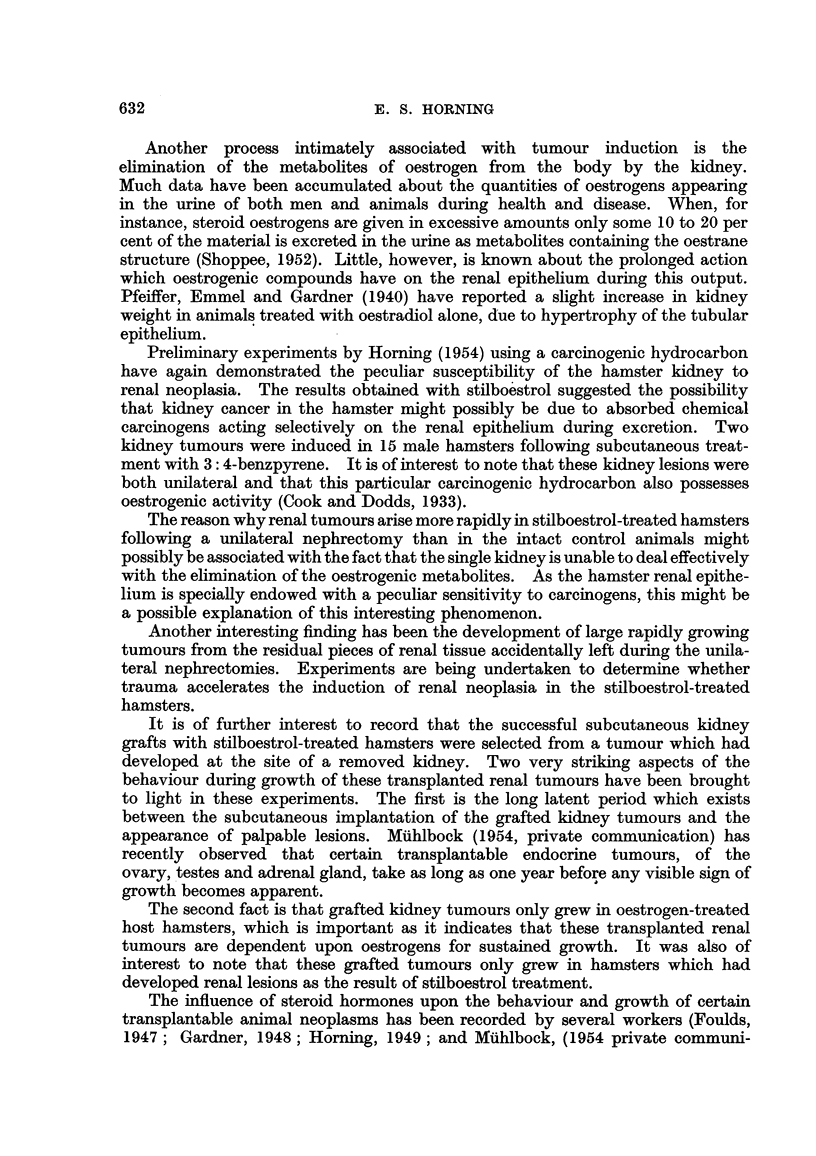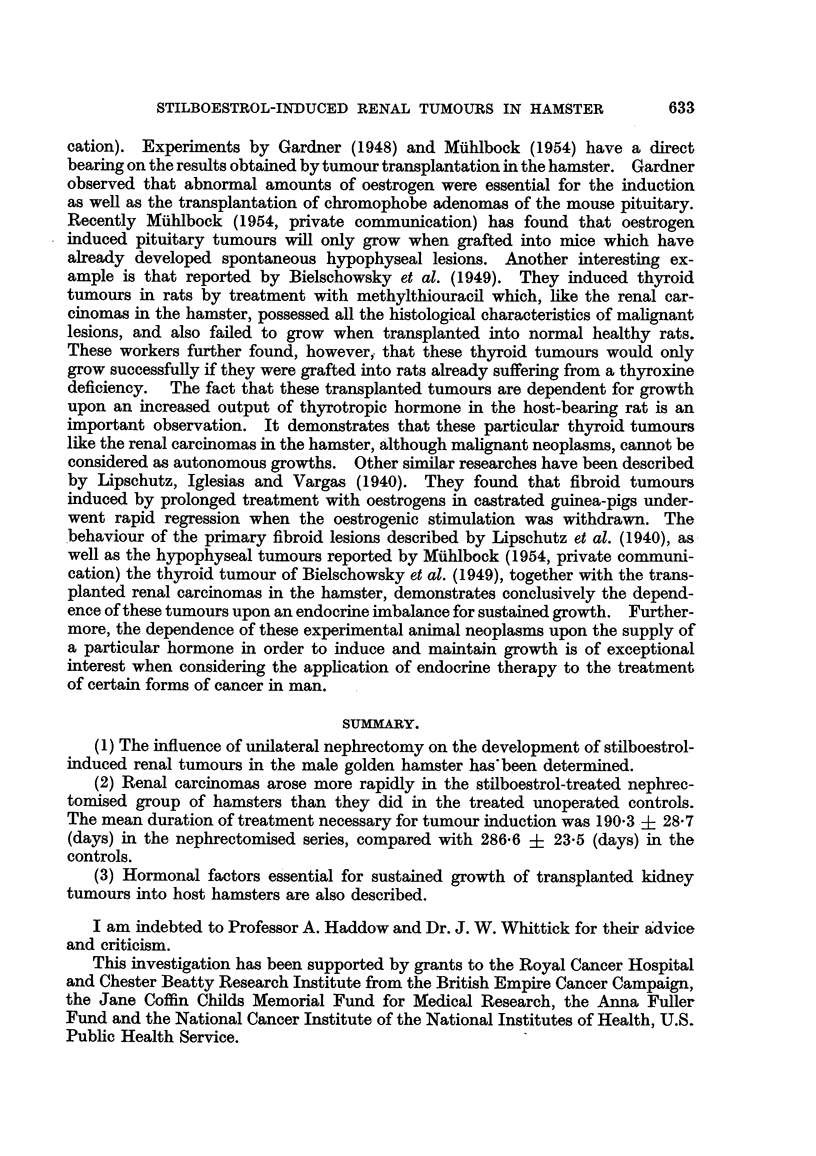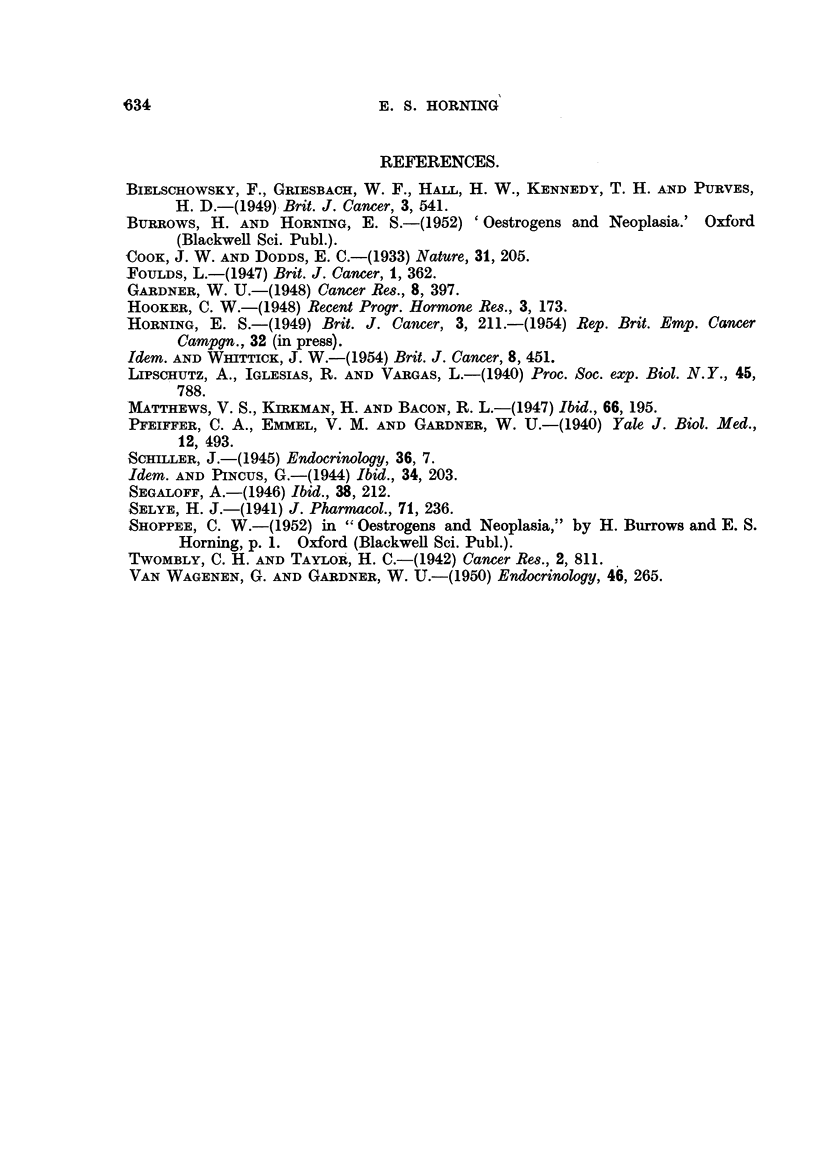# The Influence of Unilateral Nephrectomy on the Development of Stilboestrol-induced Renal Tumours in the Male Hamster

**DOI:** 10.1038/bjc.1954.68

**Published:** 1954-12

**Authors:** E. S. Horning

## Abstract

**Images:**


### THE INFLUENCE OF UNILATERAL NEPHRECTOMY ON THE DEVELOPMENT OF STILBOESTROL-INDUCED RENAL TUMOURS IN THE MALE HAMSTER.

E. S. HORNING.

*From the Chester Beatty Research Institute, Institute of Cancer Research: The Royal Cancer Hospital, London, S.W.3.*

Received for publication October 14, 1954.

IN a previous publication Horning and Whittick (1954) described the histogenesis of stilboestrol-induced renal tumours in the intact male golden hamster, the development of which was first discovered by Matthews, Kirkman and Bacon (1947). The object of this present communication is to report the influence of unilateral nephrectomy combined with oestrogen treatment, on the development and growth-rate of these tumours.

Hormonal factors determining the successful transplantation of these renal neoplasms are also described.

### MATERIAL AND METHODS.

Male golden hamsters of indeterminate ancestry bred in these laboratories were used exclusively in these experiments.

Sixty-five hamsters, all approximately 6 to 7 weeks of age, had their left kidneys removed under anaesthesia. In every instance care was taken during the nephrectomy not to damage the adrenal glands, which in the hamster lie in close contact with the kidney. Before surgical removal the renal artery, renal vein and ureter were ligatured and severed. No animals died during or immediately following the operations. Within 4 to 5 weeks after the nephrectomies were completed, 21 hamsters 10–12 weeks of age were selected at random and each received a subcutaneous implant of a 20 mg. pellet of pure diethylstilboestrol in the region of their left flank. Two hamsters of this group died on the 51st and 58th day respectively after oestrogen treatment had commenced. Death in both instances was due to enteritis. The controls for these experiments consisted of two groups. One was composed of the remaining 12 hamsters which had undergone a unilateral nephrectomy and were kept untreated. The other group consisted of 21 intact normal male hamsters, 10–12 weeks of age, all of which received subcutaneous implants of stilboestrol pellets of the same weight as those in the nephrectomised group.

All tissues were fixed either in alcoholic or aqueous Bouin and were stained either with haematoxylin and eosin or with a modification of Masson's light green.

### OBSERVATIONS.

### *Macroscopic description.*

Examination of Table I reveals that the stilboestrol-treated nephrectomised hamsters developed neoplasia much earlier than those in the treated intact control group and that the difference between the mean durations of treatment of the experimental and control series is highly significant. Even those nephrectomised hamsters which developed enteritis and had to be sacrificed on the 124th, 133rd and 134th day respectively after commencement of oestrogen treatment were found to possess small cortical lesions in their remaining kidneys. None of these small kidney tumours was palpable, and their presence was only determined at post mortem. Small kidney tumours were palpable in the nephrectomised series of hamsters as early as the 180th day after treatment, whereas the earliest renal lesion that could be palpated in the unoperated control animals was on the 260th day after stilboestrol administration. Renal tumours subsequently arose in every hamster in the control intact group as well as those in the operated series, with the exception of the two which died on the 51st and 58th day of treatment (Table I).

TABLE I.—*Development of Stilboestrol-induced Renal Tumours in Intact and Nephrectomised Hamsters.*

| Intact male hamsters. | | Unilateral nephrectomised male hamsters. | |
|---|---|---|---|
| Duration of treatment with 20 mg. stilboestrol (days). | Type of tumour obtained. | Duration of treatment with 20 mg. stilboestrol (days). | Type of tumour obtained. |
| 260 | Renal carcinoma | 133 | Sub-capsular foci |
| 265 | ,, ,, | 134 | ,, ,, |
| 272 | ,, ,, | 124 | ,, ,, |
| 275 | ,, ,, | 210 | Renal carcinoma |
| 280 | ,, ,, | 208 | ,, ,, |
| 265 | ,, ,, | 214 | ,, ,, |
| 268 | ,, ,, | 213 | ,, ,, |
| 270 | ,, ,, | 213 | ,, ,, |
| 268 | ,, ,, | 213 | ,, ,, |
| 266 | ,, ,, | 220 | ,, ,, |
| 268 | ,, ,, | 212 | ,, ,, |
| 321 | ,, ,, | 190 | ,, ,, |
| 321 | ,, ,, | 182 | ,, ,, |
| 320 | ,, ,, | 180 | ,, ,, |
| 290 | ,, ,, | 191 | ,, ,, |
| 275 | ,, ,, | 193 | ,, ,, |
| 270 | ,, ,, | 193 | ,, ,, |
| 320 | ,, ,, | 182 | ,, ,, |
| 325 | ,, ,, | 210 | ,, ,, |
| 319 | ,, ,, | * | ,, ,, |
| 302 | ,, ,, | * | |
| Mean = 286·6 ± 23·5 | | Mean = 190·3 ± 28·7 | |

* Two hamsters of this group died on the 51st and 58th day respectively after oestrogen treatment had commenced Death in both instances was attributed to enteritis.

In the early stages of development the tumours were cortical in position. They were distributed through all levels of the cortex and those which were subcapsular in position projected from the surface of the kidney (Fig. 1, 2 and 3). The size of the lesions did not always depend upon the duration of stilboestrol treatment.

The kidney tumours in both the control and experimental groups of hamsters had the same macroscopical appearance. The renal lesions in the control group were in every instance both bilateral and multifocal. In the nephrectomised group the remaining single kidney likewise bore multifocal tumours (Fig 1 and 3), and these were similar in naked-eye appearance to those in the unoperated series.

In three instances renal tumours developed on the 213th, 214th and 224th day respectively after treatment (Table I), from residual renal tissue which had been accidentally left during nephrectomy (Fig. 2 and 4). Two of these hamsters developed large tumours which occupied most of the abdominal cavities (Fig. 4). There were no peritoneal metastases in either of these animals, nor were there any secondary growths in the lymph-nodes, lungs or liver.

*Microscopic description.*

Horning and Whittick (1954) have previously shown that the large multiple tumour deposits in stilboestrol-treated male hamsters appear to be due to a rupture of anteriorly situated renal tumours, the cells of which had become implanted on the peritoneum. No metastases, however, developed in any of the treated control hamsters. Microscopically these renal tumours which arose in the nephrectomised animals were similar in their histology to those bilateral lesions which developed in the unoperated controls.

As the histogenesis of these stilboestrol-induced renal tumours was recently described in detail by Horning and Whittick (1954), it will only be briefly referred to in this communication.

The tumour foci arise from the epithelium of cortical tubules and grow both by expansion and peripheral infiltration. Proliferation from medullary tubules was not encountered. These kidney tumours are carcinomatous without any structural demarcation between the earliest hyperplastic foci and final malignant states. Well-established tumours consist of sheets and cords of compactly grouped cells of uniform appearance, solid acinar cell groups bounded by capillaries, or pseudo-glandular structures produced by cubical or columnar cells arranged in palisade fashion about capillaries. A papillary arrangement also occurs, but tubular differentiation is infrequent. In some lesions the tumour cells are spindle-shaped often producing a sarcoma-like appearance.

*Hormonal factors determining successful transplantation.*

Many unsuccessful attempts have been made to transplant these induced renal tumours subcutaneously into intact hamsters of both sexes and of varying ages. Metastatic nodules from the peritioneal surface of the body-wall, diaphragm, liver and ascending colon were also grafted both subcutaneously and intraperitoneally, but failed to grow in every instance.

All hamsters bearing tumour grafts were kept alive for a period of six months. At the end of that period they were killed and post mortem examination showed that the grafted tumour material had in every instance been absorbed by the connective tissues of the host.

The failure of these kidney tumours to grow as either subcutaneous or intra-peritoneal grafts was surprising, since they possess all the histological criteria of malignant lesions. Consideration was then given to the fact that as these neo-plasms are dependent upon high levels of oestrogen for their induction, they might also be dependent upon the continued presence of this hormone in excessive amounts for sustained growth as transplants. Consequently a large malignant lesion which developed in a nephrectomised hamster from a residual piece of renal tissue left behind during the operation was selected for transplantation (Fig. 2). Subsequently, histological examination of this growth showed it to be a clear-celled renal carcinoma in which mitoses were numerous (Fig. 7). This tumour was grafted subcutaneously into intact normal male hamsters, 3 months of age, all of which had received a 20 mg. stilboestrol pellet 12 weeks previous to transplantation. Two of these oestrogen-treated hamsters bearing grafts unfortunately died within 3 weeks of the operation. Twenty untreated male hamsters of a similar age received grafts of the same tumour, and were kept as controls.

Small palpable tumours began to appear after $5\frac{1}{2}$ months in 4 out of the 8 remaining oestrogen-treated animals. No tumour nodules could be detected in any of the control untreated hamsters which had received tumour grafts.

Six months after transplantation the hamster bearing the largest of the four subcutaneous-grafted tumours was killed. At post-mortem this tumour was found to be approximately $1\frac{3}{4}$ in. in length and $\frac{3}{4}$ in. across. The stilboestrol pellet was seen embedded in the connective tissue adjacent to the tumour (Fig. 5).

This tumour was grafted subcutaneously into oestrogen-treated hamsters only, as it had previously failed to grow when transplanted into normal untreated animals. After a portion of the grafted tumour had been fixed and the rest transplanted, the abdominal cavity of the host-bearing hamster was opened up. It was then found that each kidney had developed multifocal sub-capsular lesions as the result of stilboestrol treatment (Fig. 6).

Histological examination of the first generation of this grafted tumour showed it to be an actively growing, clear-celled carcinoma with numerous mitoses. It was also very similar in structure to the original primary tumour. During the following 3 weeks the remaining hamsters bearing the first generation of successfully growing subcutaneous tumour grafts were sacrificed. Portions of the grafted tumour were likewise transplanted into hamsters which had previously been treated with stilboestrol. Every hamster bearing tumour grafts was found at

---

EXPLANATION OF PLATES.

FIG. 1.—Multifocal renal tumours in the remaining kidney of a nephrectomised stilboestrol-treated male hamster. $\times 1\frac{1}{2}$.

FIG. 2.—Large renal carcinoma which developed from a residual piece of kidney accidentally left during a unilateral nephrectomy. The remaining intact kidney has developed sub-capsular tumours in response to stilboestrol treatment. $\times 1\frac{1}{2}$.

FIG. 3.—Stilboestrol induced multifocal renal lesions which arose in the remaining kidney of a nephrectomised hamster. $\times 1\frac{1}{2}$.

FIG. 4.—Large renal tumour occupying most of the abdominal cavity in a nephrectomised stilboestrol-treated male hamster. The remaining kidney is obscured from view by the large carcinoma which arose from a piece of kidney tissue accidentally left during a nephrectomy. $\times 2$.

FIG. 5.—Transplanted kidney tumour growing subcutaneously in a male hamster which had been pre-treated with stilboestrol. $\times \frac{5}{8}$.

FIG. 6.—The same as in Fig. 5. The grafted subcutaneous tumour has been removed. The abdominal wall has been cut away exposing the two kidneys, both of which have developed cortical lesions in response to stilboestrol treatment. $\times \frac{5}{8}$.

FIG. 7.—Section of primary renal carcinoma, part of which was successfully grafted subcutaneously into a male hamster which had been pre-treated with stilboestrol. $\times 325$.

FIG. 8.—First generation of serial kidney transplant seen in Fig. 5 and 6. Observe the numerous mitoses, and compare with section of primary renal carcinoma seen in Fig. 7. $\times 325$.

1 2 3 4

Horning.

5

6

7

8

post mortem to have primary bilateral and multifocal kidney lesions. The dependence of these transplanted kidney tumours, which are now in their second generation of serial transplantation, upon oestrogen for sustained growth is still being investigated.

### DISCUSSION.

In a recent communication Horning and Whittick (1954), working on the histogenesis of experimental neoplasia in the intact male hamster, confirmed the interesting observation first described by Matthews, Kirkman and Bacon (1947) that prolonged stilboestrol administration induces kidney tumours in these rodents. This present investigation on the effects of unilateral nephrectomy on the development and growth-rate of these kidney lesions, together with the hormonal factors influencing transplantation, is an extension of this work. These results have given some additional information on the mechanism of renal tumorigenesis in oestrogen-treated hamsters.

It has been conclusively shown that renal neoplasia develops more rapidly in unilaterally nephrectomised hamsters than it does in the intact controls, and furthermore that the difference between the mean durations of treatment of the control and the experimental groups is highly significant.

Why the kidney epithelium of the normal intact male hamster should possess this peculiar susceptibility to develop kidney tumours following oestrogen administration, while other species of rodents never develop kidney lesions after similar treatment, has never been fully explained, nor has it been clearly understood why only males develop kidney tumours under these conditions and the females never do. Burrows and Horning (1952) are of the opinion that exposure of the female to plentiful amounts of oestrogen during life may possibly cause some degree of physiological adaptation.

When considering the problem of the induction of kidney neoplasia in stilboestrol-treated hamsters, it should be remembered that tumour formation under these conditions is intimately associated both with the capacity of the liver to inactivate oestrogens circulating in the blood stream and that of the kidney in aiding their elimination from the body. Although the liver is the principal site of oestrogen inactivation, it has been shown by Schiller (1945) that oestrogens are also inactivated by the rat kidney.

The capacity of the liver to inactivate oestrogens varies considerably in different animals. Twombly and Taylor (1942) have demonstrated that slices of human liver do so more slowly than rat livers; and Van Wagenen and Gardner (1950) have observed that in primates the liver failed to reduce the potency of oestrogen.

It might be possible that the hamster liver is not endowed with such a high capacity as that of the rat and other rodents for inactivating oestrogens, and this might be one of the reasons why hamsters develop renal neoplasia and other species of rodents do not.

Experiments are being undertaken to excise a large portion of the liver in living hamsters before stilboestrol treatment has commenced, as it has been shown by several workers that this operation correspondingly diminishes the inactivation of oestrogen (Selye, 1941; Schiller and Pincus, 1944 ; and Segaloff, 1946). If this is so, this form of treatment should accelerate the induction of renal carcinoma and so give more insight into the rôle played by the hamster liver in renal carcinogenesis.

Another process intimately associated with tumour induction is the elimination of the metabolites of oestrogen from the body by the kidney. Much data have been accumulated about the quantities of oestrogens appearing in the urine of both men and animals during health and disease. When, for instance, steroid oestrogens are given in excessive amounts only some 10 to 20 per cent of the material is excreted in the urine as metabolites containing the oestrane structure (Shoppee, 1952). Little, however, is known about the prolonged action which oestrogenic compounds have on the renal epithelium during this output. Pfeiffer, Emmel and Gardner (1940) have reported a slight increase in kidney weight in animals treated with oestradiol alone, due to hypertrophy of the tubular epithelium.

Preliminary experiments by Horning (1954) using a carcinogenic hydrocarbon have again demonstrated the peculiar susceptibility of the hamster kidney to renal neoplasia. The results obtained with stilboestrol suggested the possibility that kidney cancer in the hamster might possibly be due to absorbed chemical carcinogens acting selectively on the renal epithelium during excretion. Two kidney tumours were induced in 15 male hamsters following subcutaneous treatment with 3:4-benzpyrene. It is of interest to note that these kidney lesions were both unilateral and that this particular carcinogenic hydrocarbon also possesses oestrogenic activity (Cook and Dodds, 1933).

The reason why renal tumours arise more rapidly in stilboestrol-treated hamsters following a unilateral nephrectomy than in the intact control animals might possibly be associated with the fact that the single kidney is unable to deal effectively with the elimination of the oestrogenic metabolites. As the hamster renal epithelium is specially endowed with a peculiar sensitivity to carcinogens, this might be a possible explanation of this interesting phenomenon.

Another interesting finding has been the development of large rapidly growing tumours from the residual pieces of renal tissue accidentally left during the unilateral nephrectomies. Experiments are being undertaken to determine whether trauma accelerates the induction of renal neoplasia in the stilboestrol-treated hamsters.

It is of further interest to record that the successful subcutaneous kidney grafts with stilboestrol-treated hamsters were selected from a tumour which had developed at the site of a removed kidney. Two very striking aspects of the behaviour during growth of these transplanted renal tumours have been brought to light in these experiments. The first is the long latent period which exists between the subcutaneous implantation of the grafted kidney tumours and the appearance of palpable lesions. Mühlbock (1954, private communication) has recently observed that certain transplantable endocrine tumours, of the ovary, testes and adrenal gland, take as long as one year before any visible sign of growth becomes apparent.

The second fact is that grafted kidney tumours only grew in oestrogen-treated host hamsters, which is important as it indicates that these transplanted renal tumours are dependent upon oestrogens for sustained growth. It was also of interest to note that these grafted tumours only grew in hamsters which had developed renal lesions as the result of stilboestrol treatment.

The influence of steroid hormones upon the behaviour and growth of certain transplantable animal neoplasms has been recorded by several workers (Foulds, 1947; Gardner, 1948; Horning, 1949; and Mühlbock, (1954 private communication). Experiments by Gardner (1948) and Mühlbock (1954) have a direct bearing on the results obtained by tumour transplantation in the hamster. Gardner observed that abnormal amounts of oestrogen were essential for the induction as well as the transplantation of chromophobe adenomas of the mouse pituitary. Recently Mühlbock (1954, private communication) has found that oestrogen induced pituitary tumours will only grow when grafted into mice which have already developed spontaneous hypophyseal lesions. Another interesting example is that reported by Bielschowsky *et al.* (1949). They induced thyroid tumours in rats by treatment with methylthiouracil which, like the renal carcinomas in the hamster, possessed all the histological characteristics of malignant lesions, and also failed to grow when transplanted into normal healthy rats. These workers further found, however, that these thyroid tumours would only grow successfully if they were grafted into rats already suffering from a thyroxine deficiency. The fact that these transplanted tumours are dependent for growth upon an increased output of thyrotropic hormone in the host-bearing rat is an important observation. It demonstrates that these particular thyroid tumours like the renal carcinomas in the hamster, although malignant neoplasms, cannot be considered as autonomous growths. Other similar researches have been described by Lipschutz, Iglesias and Vargas (1940). They found that fibroid tumours induced by prolonged treatment with oestrogens in castrated guinea-pigs underwent rapid regression when the oestrogenic stimulation was withdrawn. The behaviour of the primary fibroid lesions described by Lipschutz *et al.* (1940), as well as the hypophyseal tumours reported by Mühlbock (1954, private communication) the thyroid tumour of Bielschowsky *et al.* (1949), together with the transplanted renal carcinomas in the hamster, demonstrates conclusively the dependence of these tumours upon an endocrine imbalance for sustained growth. Furthermore, the dependence of these experimental animal neoplasms upon the supply of a particular hormone in order to induce and maintain growth is of exceptional interest when considering the application of endocrine therapy to the treatment of certain forms of cancer in man.

SUMMARY.

(1) The influence of unilateral nephrectomy on the development of stilboestrol-induced renal tumours in the male golden hamster has been determined.

(2) Renal carcinomas arose more rapidly in the stilboestrol-treated nephrectomised group of hamsters than they did in the treated unoperated controls. The mean duration of treatment necessary for tumour induction was $190 \cdot 3 \pm 28 \cdot 7$ (days) in the nephrectomised series, compared with $286 \cdot 6 \pm 23 \cdot 5$ (days) in the controls.

(3) Hormonal factors essential for sustained growth of transplanted kidney tumours into host hamsters are also described.

I am indebted to Professor A. Haddow and Dr. J. W. Whittick for their advice and criticism.

This investigation has been supported by grants to the Royal Cancer Hospital and Chester Beatty Research Institute from the British Empire Cancer Campaign, the Jane Coffin Childs Memorial Fund for Medical Research, the Anna Fuller Fund and the National Cancer Institute of the National Institutes of Health, U.S. Public Health Service.

### REFERENCES.

BIELSCHOWSKY, F., GRIESBACH, W. F., HALL, H. W., KENNEDY, T. H. AND PURVES, H. D.—(1949) *Brit. J. Cancer*, **3**, 541.
BURROWS, H. AND HORNING, E. S.—(1952) 'Oestrogens and Neoplasia.' Oxford (Blackwell Sci. Publ.).
COOK, J. W. AND DODDS, E. C.—(1933) *Nature*, **31**, 205.
FOULDS, L.—(1947) *Brit. J. Cancer*, **1**, 362.
GARDNER, W. U.—(1948) *Cancer Res.*, **8**, 397.
HOOKER, C. W.—(1948) *Recent Progr. Hormone Res.*, **3**, 173.
HORNING, E. S.—(1949) *Brit. J. Cancer*, **3**, 211.—(1954) *Rep. Brit. Emp. Cancer Campgn.*, **32** (in press).
*Idem.* AND WHITTICK, J. W.—(1954) *Brit. J. Cancer*, **8**, 451.
LIPSCHUTZ, A., IGLESIAS, R. AND VARGAS, L.—(1940) *Proc. Soc. exp. Biol. N.Y.*, **45**, 788.
MATTHEWS, V. S., KIRKMAN, H. AND BACON, R. L.—(1947) *Ibid.*, **66**, 195.
PFEIFFER, C. A., EMMEL, V. M. AND GARDNER, W. U.—(1940) *Yale J. Biol. Med.*, **12**, 493.
SCHILLER, J.—(1945) *Endocrinology*, **36**, 7.
*Idem.* AND PINCUS, G.—(1944) *Ibid.*, **34**, 203.
SEGALOFF, A.—(1946) *Ibid.*, **38**, 212.
SELYE, H. J.—(1941) *J. Pharmacol.*, **71**, 236.
SHOPPEE, C. W.—(1952) in "Oestrogens and Neoplasia," by H. Burrows and E. S. Horning, p. 1. Oxford (Blackwell Sci. Publ.).
TWOMBLY, C. H. AND TAYLOR, H. C.—(1942) *Cancer Res.*, **2**, 811.
VAN WAGENEN, G. AND GARDNER, W. U.—(1950) *Endocrinology*, **46**, 265.